# Microvascular benefits of hypertension and glucose control in type 2 diabetes

**Published:** 2008

**Authors:** BWJ Van Rensburg

**Affiliations:** Division of Nephrology, University of the Free State, Bloemfontein

When albumin starts leaking through the glomerular tuft of capillaries into the urine of diabetic patients, it is an indication of small-blood vessel disease throughout the body. The more protein found in the urine, the more damage is evident elsewhere (retinopathy and neuropathy).^1^ Our best weapon in primarily preventing this damage has been tight blood glucose control, from as early after the diagnosis of diabetes as possible, and we have good evidence of its success in type 1 diabetics.

Type 2 diabetics are more likely to have macrovascular disease earlier in the course of their disease. At this stage, we believe that the pathogenesis of microvascular complications is likely to be similar in both type 1 and type 2 diabetics, but the exact time of onset of type 2 diabetes is often unclear. By the time patients first present to a medical practitioner, many type 2 diabetics may already have evidence of microvascular disease, as manifested by the presence of microalbuminuria.^2^ This has made the study of primary prevention of microvascular disease in type 2 diabetics more difficult.

Once microalbuminuria is present, secondary prevention strategies, in addition to glucose control, include blocking the renin–angiotensin system, irrespective of the presence of hypertension. Recommendations often include a combination of both an ACE inhibitor (ACEI) and angiotensin receptor blocker (ARB) if the target reduction in proteinuria cannot be achieved with either alone.

Once hypertension and macroalbuminuria (overt proteinuria) become evident, blood pressure control takes centre stage, targeting lower levels than in the general population,^3^ while still persevering with glucose control and blocking the renin–angiotensin system. Just as it takes time for diabetic complications to become manifest, it has become clear that it takes time to reverse the damage with interventions.

An interesting documentation of this fact was illustrated with serial native renal biopsies of patients rendered euglycaemic with pancreas transplantation.^4^ No significant difference in histological measurements of diabetic nephropathy were present after five years, but at 10 years it was evident that significant regression had occurred. This fact may be important in evaluating negative outcomes in trials lasting less than five years. When using interventions, it is important to try and achieve optimal targets. Currently a glycated haemoglobin of below 7%, blood pressure of less than 130/80 mmHg and proteinuria of less than 500–1 000 mg/day are recommended.^3^

Nephrologists have used proteinuria as a surrogate marker for interventional studies in much the same way as cardiologists use left ventricular hypertrophy, with the same questions regarding the correlation with hard endpoints such as end-stage renal disease or all-cause mortality. During 2008, several studies have become available that may impact on our strategies for the management of microvacular complications in type 2 diabetics.

Both the ACCORD^5^ and ADVANCE^6^ (low glucose arm) trials targeted a lower HbA_1c_ level in type 2 diabetics. The ACCORD study aimed for a glycated haemoglobin level of below 6% (but attained 6.4%) in a high cardiovascular risk group that included patients with microalbuminuria. The trial was stopped after a mean of 3.5 years of follow up due to a relative increase in mortality of 22% in the intensive glucose-lowering arm. The ADVANCE trial similarly studied type 2 diabetics with high cardiovascular risk, including about 27% with a history of microalbuminuria. The study did not reveal a significant difference in cardiovascular complications after a mean follow-up period of five years, but clearly demonstrated a 21% relative reduction in nephropathy. Both studies, therefore, failed to demonstrate benefit from tight glucose control on macrovascular disease for the period of the study. Possible causes for the worse outcome in the ACCORD study have been postulated to be more rapid glucose control, weight gain and the different drugs used to control blood sugar.^7^

Another study that may be of concern is ONTARGET,^8^ which found a significantly higher adverse-event rate with the use of combined ACEI and ARB, compared to each alone, in high cardiovascular risk patients (36% were diabetics and 13% had microalbuminuria). In particular, significantly more renal dysfunction and a trend towards more end-stage renal disease requiring dialysis were found in the combined ACEI and ARB arm.

From these recent studies, the logical question therefore arises: when we intervene later in a type 2 diabetic patient’s course of disease when macrovascular disease is more prominent, are we not substituting the benefit we gained from improvement in microvascular disease, with more cardiovascular events from the aggressive targets currently investigated? Should we stratify patients and proceed more cautiously in those with established disease, while reserving very aggressive targets for early diabetics?

Clearly, final answers to these questions will require further study, but part of the answer may be contained in the follow-up report of the STENO-2 trial.^9^ One hundred and sixty white Danish patients with type 2 diabetes and microalbuminuria were studied in an intensive, multifactorial intervention trial, targeting a glycated haemoglobin of less than 6.5%, fasting serum cholesterol below 4.5 mmol/l, fasting serum triglycerides less than 1.7 mmol/l, a systolic blood pressure of less than 130 mmHg and a diastolic blood pressure of less than 80 mmHg. Patients were treated with blockers of the renin–angiotensin system regardless of blood pressure, and received low-dose aspirin as primary prevention and focused behaviour modification.

These interventions were compared to conventional treatment and after 7.8 years, a 50% reduction in cardiovascular and microvascular endpoints were noted. The patients and their doctors were instructed about the benefits of the intensive therapy and after another 5.5 years (13.3 years in total), a sustained benefit was shown with respect to death from any cause and from cardiovascular causes in the intensive-control arm.

Diabetic nephropathy is currently the most important cause of end-stage renal failure in communities living a western lifestyle. In South Africa it is expected to rapidly increase and with the high cost of renal replacement therapy, a significant group of patients with end-stage renal failure due to diabetic nephropathy will die without the chance of dialysis or transplantation. It is the duty of every healthcare worker in our country to try and prevent or delay this tragic complication in our patients.

The current studies demonstrate that intensive glucose control to a target of glycated haemoglobin below 6.5% does have benefit with regard to microvascular disease, and that it should form part of a multifactorial intervention aimed at reducing overall cardiovascular risk. The lower target has not been accepted in guidelines at this stage, and caution should be exercised in patients with high cardiovascular risk in achieving targets too rapidly. Likewise, a blood pressure reduction below 110/75 mmHg may increase risk in type 2 diabetics with ischaemic heart disease, and the combination of ACEI and ARB in the aggressive reduction of proteinuria in these patients needs further clarification.
